# Advances in Drug Targeting, Drug Delivery, and Nanotechnology Applications: Therapeutic Significance in Cancer Treatment

**DOI:** 10.3390/pharmaceutics17010121

**Published:** 2025-01-16

**Authors:** Fatih Ciftci, Ali Can Özarslan, İmran Cagri Kantarci, Aslihan Yelkenci, Ozlem Tavukcuoglu, Mansour Ghorbanpour

**Affiliations:** 1 Department of Biomedical Engineering, Faculty of Engineering, Fatih Sultan Mehmet Vakıf University, Istanbul 34015, Turkey; 2 Department of Technology Transfer Office, Fatih Sultan Mehmet Vakıf University, Istanbul 34015, Turkey; 3 Department of Metallurgical and Materials Engineering, Istanbul University-Cerrahpasa, Istanbul 34320, Turkey; alicanozarslan@gmail.com; 4 Department of Bioengineering, Faculty of Chemistry-Metallurgy, Yildiz Technical University, Istanbul 34210, Turkey; cagri_kantarci@hotmail.com; 5 Department of Pediatric Dentistry, Faculty of Dentistry, University of Health Sciences, Istanbul 34668, Turkey; aslihanzihni@gmail.com; 6 Department of Biochemistry, Faculty of Hamidiye Pharmacy, University of Health Sciences, Istanbul 34668, Turkey; ozlemoztolan@gmail.com; 7 Department of Medicinal Plants, Faculty of Agriculture and Natural Resources, Arak University, Arak 38156-8-8349, Iran; m-ghorbanpour@araku.ac.ir

**Keywords:** drug carriers, drug delivery systems, drug targeting, nanotechnology

## Abstract

In the 21st century, thanks to advances in biotechnology and developing pharmaceutical technology, significant progress is being made in effective drug design. Drug targeting aims to ensure that the drug acts only in the pathological area; it is defined as the ability to accumulate selectively and quantitatively in the target tissue or organ, regardless of the chemical structure of the active drug substance and the method of administration. With drug targeting, conventional, biotechnological and gene-derived drugs target the body’s organs, tissues, and cells that can be selectively transported to specific regions. These systems serve as drug carriers and regulate the timing of release. Despite having many advantageous features, these systems have limitations in thoroughly treating complex diseases such as cancer. Therefore, combining these systems with nanoparticle technologies is imperative to treat cancer at both local and systemic levels effectively. The nanocarrier-based drug delivery method involves encapsulating target-specific drug molecules into polymeric or vesicular systems. Various drug delivery systems (DDS) were investigated and discussed in this review article. The first part discussed active and passive delivery systems, hydrogels, thermoplastics, microdevices and transdermal-based drug delivery systems. The second part discussed drug carrier systems in nanobiotechnology (carbon nanotubes, nanoparticles, coated, pegylated, solid lipid nanoparticles and smart polymeric nanogels). In the third part, drug targeting advantages were discussed, and finally, market research of commercial drugs used in cancer nanotechnological approaches was included.

## 1. Introduction

The majority of commonly used drugs do not exert their activity in the body by selective distribution in pathological organs, cells or tissues [1,2,3,4]. Generally, these drugs prefer to be distributed throughout the body. Moreover, drugs have to cross many biological barriers such as organs, cells and intracellular compartments to reach the site of action [2,5,6]. Meanwhile, drugs may accumulate in normal organs and tissues that are not involved in the pathological process. This results in the need for large amounts to be taken by the patient in order to reach therapeutic concentrations in the required body compartments, as well as many negative side effects. Drug targeting offers a solution to all these problems. Drug targeting is the ability of a drug to selectively and quantitatively accumulate in the target tissue or organ, independent of the chemical structure and mode of uptake of the active substance. In this way, the concentration of the drug will be high in diseased areas and will remain at a minimum level in other areas to prevent negative side effects [7,8]

Transport of the drug into the structure where it will act is one of the main problems in the pharmaceutical and biotechnological industries [9,10]. Therefore, drug delivery systems have always been an area of interest for researchers. New developments in biotechnology and research in other related sciences have helped in the discovery and rational design of many new drugs. However, most drugs are constrained by poor solubility, high toxicity, high dose, accumulation of drug due to poor solubility, nonspecific transport, in vivo degradation and short half-life [10,11,12,13,14]. Nowadays, many researchers from various disciplines are involved in the development of specific new drug delivery systems in order to minimize the increasing problems of drugs and to transform new developments into clinical efficacy. Targeted drug delivery is defined as the specific release of a bioactive agent into a certain structure at a certain rate. Targeted drug delivery systems deliver drugs more efficiently and practically than current drugs, improve patient compliance, prolong drug half-life and reduce healthcare costs. Therefore, the development of techniques that can selectively deliver drugs to pathologic cells, tissues or organs is one of the most important areas of drug research today.

Advances in nanotechnology have had a significant impact on the pharmaceutical industry, especially nanoparticles (NPs), which have many applications in the clinic. Nanotechnology focuses on the formulation of therapeutic agents in liposomes and nanocarriers (nanoparticles, nanocapsules, micelles and dendrimers, etc.). These formulations enable targeted drug delivery to the diseased structure. Since nanoparticles have the potential to be used in the diagnosis and treatment of many diseases, it is conceivable that they will play a greater role in drug delivery system technology in the near future. The process of nanotechnology applications in various branches of science, especially in the field of health, becoming widespread and new drugs replacing traditional drugs is accelerating. In this process, nanotechnology and biotechnology pave the way for the development of numerous drugs produced by pharmaceutical industries. Various active substance delivery systems and targeting systems have been developed to minimize active substance degradation and loss, prevent harmful side effects, and increase bioavailability and potency. Some of these systems include liposomes, nanoparticles, active substance-polymer conjugates and polymeric micelles [7,8,15,16,17,18,19].

The process of administering pharmaceuticals to humans or animals in order to achieve a therapeutic effect is known as drug delivery [20,21]. The pharmaceutical delivery system typically contains an appropriate dosage form that delivers the medication to the body; a mechanism for releasing the medication from the dosage form to the target cells following administration; and the device or technique used to create the dosage form [22,23,24]. Over the years, controlled drug delivery systems which dispense medications at predetermined rates for predetermined amounts of time have been developed. Unlike traditional dosage forms, controlled drug delivery systems prevent the drug from being released immediately and cause drug levels in the blood to fluctuate depending on the dosage form [25,26,27,28]. But it would be ideal if medications could be given in a way that perfectly meets the needs at the right periods and at the right location. It would have been very helpful if the active medicines could be administered through a system that detected the disease symptom, assessed its strength, and lastly acted to deliver the appropriate dosage of medication in response. The development of a smart drug delivery system like this would necessitate some kind of feedback mechanism to link the drug administration rate with physiological need [29,30,31,32,33]. Some medications that must enter the intracellular environment in order to have the desired effects are either incapable of being absorbed by cells or are wiped out by interaction with cytosol proteins. These issues have been resolved through advancements in nanotechnology and the development of new drug carriers.

The aim of drug targeting is to selectively transport, absorb and distribute the pharmacologic agent to the site of action. With this selective targeting, undesirable side effects are reduced, optimal therapeutic response is obtained, and substances with toxic effects at high doses can be used safely. With targeting, conventional, biotechnological and gene-derived drugs can be selectively delivered to specific parts of the body such as organs, tissues and cells. In the field of drug targeting [34], the liposomal formulation Doxil^®^ (Doxurobicin, Baxter Healthcare Corporation, Deerfield, IL, USA) [35] and the nanoparticle formulation Abraxane^®^ (Paclitaxel, Abraxis BioScience, Los Angeles, CA, USA) [36] have been approved by the US Food and Drug Administration (FDA) as new drug delivery systems. In addition, tumor targeting studies with specific monoclonal antibodies are currently being conducted. Monoclonal antibodies such as Erbitux^®^ (Cetuximab, Eli Lilly and Company, Indianapolis, IN, USA) [37,38] used in colorectal cancer treatment, Vectibix^®^ (Panitumumumab, Amgen Inc., Thousand Oaks, CA, USA) [39] and Trastuzumab (Herceptin^®^, Genentech, South San Francisco, CA, USA) [40] used in antiangiogenic therapy; small molecule tyrosine kinase inhibitors such as Imatinib (Gleevec^®^, Novartis Pharmaceuticals Corporation, Basel, Switzerland) [41], Erlotinib (Tarveca^®^, CHEPLAPHARM, Ziegelhof, Germany) [42], Sorafenib (Nexavar^®^, Bayer HealthCare Pharmaceuticals Inc., Whippany, Hanover, NJ, USA) [43,44], and Sunitinib (Sutent^®^, Pfizer, Hudson Boulevard East New York, NY, USA) [45] have received FDA approval and are used in the clinic.

## 2. Drug Delivery Systems

Modern drug delivery systems have revolutionized therapeutic approaches by enabling precise, controlled, and efficient delivery of pharmaceutical compounds. These systems are designed to overcome traditional drug administration limitations, such as poor bioavailability, systemic toxicity, and lack of targeted delivery. Active and passive drug delivery mechanisms play pivotal roles in ensuring effective drug transport. While passive delivery relies on natural diffusion and concentration gradients, active delivery employs external stimuli or molecular recognition to achieve targeted release, enhancing therapeutic efficacy. Hydrogel-based drug delivery systems have gained attention due to their biocompatibility, high water content, and tunable properties, making them suitable for controlled and sustained drug release applications. Thermoplastic drug delivery systems leverage the versatility of thermoplastic polymers, offering robustness, flexibility, and adaptability for fabricating drug carriers that respond to physiological conditions. Microdevice-based delivery systems represent a breakthrough in precision medicine, utilizing miniaturized devices for localized drug administration, improving therapeutic outcomes while minimizing systemic effects. Transdermal patches provide a non-invasive alternative for drug administration, ensuring sustained release through the skin barrier, which improves patient compliance and maintains steady drug plasma levels. This section delves into these advanced drug delivery modalities, highlighting their principles, advantages, and potential for addressing current challenges in pharmaceutical science.

### 2.1. Active and Passive Drug Delivery

In some cases, drug targeting can be achieved in a simple way. The drug is administered directly to the pathologic site. Some of the successful examples of this approach are direct hormonal drug administration into the joints in the treatment of arthritis and direct administration of thrombolytic enzymes used in the treatment of myocardial infarction caused by thrombus directly into the coronary vessels [46,47]. Active targeting is defined as specific interactions between the drug delivery system and target cells, in short ligand-receptor interactions [48]. The essence of active targeting is based on the use of targeted ligands such as antibodies and peptides that can bind specifically to receptor structures directed to the target structure. Examples of targeted ligands from drug delivery systems used in active targeting to tumor cells include folate, transferrin, and galactosamine [49,50]. The success of active targeting is ensured by the correct selection of targeting vehicles that show high affinity for cell surface receptors and chemical modifications to form appropriate conjugation. Active targeting can be achieved by ligand-receptor, antigen-antibody interactions or by targeting aptamers to identify pathological cells with various molecules concentrated at the pathological site [51,52,53]. Aptamers are DNA or RNA oligonucleotide sequences that bind selectively with high affinity to the target utilized in the active targeting of therapeutics [54,55]. The targeted therapeutic agent prefers high drug accumulation in the pathological structure with the help of a carrier that can combine with a cell or tissue-specific ligand. Thus, besides having the ability to combine with different targeting ligands, nanosystems of varying sizes can offer excellent opportunities for overcoming physiological barriers and efficient cellular uptake of the drug. Various nanosystems can achieve higher concentrations in cellular uptake than normal drugs [56,57].

**Figure 1:**
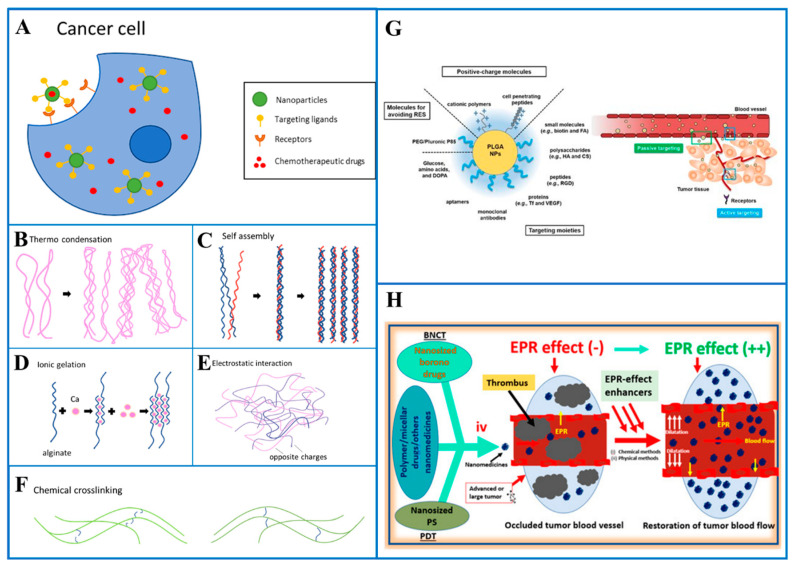
(**A**) Drug delivery with active targeting on a cancer cell, crosslinking mechanisms. (**B**) Thermo condensation, (**C**) Self-assembly, (**D**) Ionic gelation, (**E**) Electrostatic interaction, (**F**) Chemical crosslinking, (**G**) Various surface-engineered poly(lactic-co-glycolic acid) (PLGA) nanoparticles (NPs) for passive or active tumor targeting. Arg-Gly-Asp (RGD); reticuloendothelial system (RES); transferrin (Tf); vascular endothelial growth factor (VEGF) Reproduced from [71], MDPI, 2019. (**H**) Graphical abstract of enhanced permeability and retention (EPR) Reproduced from [72], MDPI, 2022.

By integrating the drug into a macromolecule or nanoparticle that enters the target tissue passively, passive targeting is accomplished [16,58,59,60]. The length of circulation is determined, and the drug is ensured to reach the target organ with a coating around the nanoparticle. The surface of the nanoparticle can be made hydrophilic by the addition of a chemical such as as polyethylene glycol (PEG) [61,62,63,64,65], which allows the H_2_O molecules to interact with the O_2_ molecules through hydrogen bonding. After this interaction, the material becomes antiphagocytic. In a study for passive or active tumor targeting, poly(lactic-co-glycolic acid) (PLGA) nanoparticles (NPs) were produced in different compositions and combinations. Arg-Gly-Asp (RGD); chitosan; dopamine (DOPA); folic acid; hyaluronic acid; poly(ethylene glycol) (PEG); reticuloendothelial system (RES); transferrin (Tf); and vascular endothelial growth factor (VEGF) were loaded onto poly(lactic-co-glycolic acid) nanoparticles (NPs), demonstrating the passive and active tumor targeting capabilities of ligand-receptor interactions (Figure 1G) [66,67,68,69]. The enhanced permeability and retention (EPR) (Figure 1H) [61,70] effect is observed in cancerous tissues due to extensive vascular leakage and lack of lymphatic drainage. This enhanced vascular permeability effect in tumor sites compared to healthy tissues can be utilized for the delivery and accumulation of passive targeting nanocarriers in targeted tissues.

Active targeting improves targeting such that a medicine becomes specialized to a target spot. The diseased tissue can be actively targeted by knowing the nature of the receptors on cells associated with it (Figure 1A). This makes the utilization of ligands, which allow the drug to specifically bind to the cells with the corresponding receptors, possible. Transferrin and RGD motif [73,74,75] are examples of cell-specific ligands which are utilized to target specific receptors on tumor cells. Magnetoliposomes, which are often used in Magnetic Resonance Imaging (MRI) as a contrast agent, can also be used in active targeting. Merging these liposomes with drugs allows magnetic placement to help the delivery of drugs to a specific area of the body. Redox potential is the basis for another triggering mechanism, hypoxia, which is one of the side effects of tumors and affects the redox potential in the tumor’s proximity. Different tumor types can be targeted specifically by particles via altering the redox potential that causes the release of the payload.

In general, passive targeting is the delivery of drugs to specific sites through natural physiological processes and factors. Passive targeting exploits anatomical differences between normal and pathological tissues to move drugs to the required site. Research has shown that in some cases (such as tumor cells) the permeability of blood vessel walls is increased. Due to the loose vascularity of the tumor, the drug delivery system spontaneously penetrates through the blood vessel walls into the interstitium. This is called the increased EPR effect (Figure 1H) [76,77,78]. The EPR effect has been observed not only in tumor cells but also in areas of inflammation. Maeda et al. demonstrated that excessive bradykinin release at sites of infection or inflammation elicits the EPR effect. The only difference between the EPR effect in an infection-based and tumor cell is the duration of drug retention time. When infection occurs in a normal cell, the retention time is shorter than in a cancer cell because the lymphatic drainage system is still functioning, so the infection can dissipate within a few days. In contrast, macromolecular or lipid drugs can take weeks to take hold in the cancer cell. The EPR effect has been utilized to transport various therapeutics to the site of action. Many studies have suggested findings supporting the mechanism of passive targeting. In the 1980s and 1990s, many nanocarriers based on passive targeting mechanisms were designed. For example, doxorubicin (DOXIL) designed in liposomal formulation was observed to be 6 times more effective compared to free doxorubicin [79,80,81].

### 2.2. Hydrogel Drug Delivery Systems

Selecting the ideal drug delivery material for the required clinical purpose might be challenging due to the diversity of the currently available polymers. Developments in polymer science resulted in the production of hydrogel systems which have been employed for different kinds of medication delivery applications over the years. Hydrogels are among the most promising biopolymers being used for drug delivery applications. They are made of tunable, injectable polymer-based networks which can absorb and hold large quantities of water. They can be produced from a variety of different polymers such as polyethylene glycol (PEG), poly(acrylic acid) (PAA), poly(N-isopropylacrylamide), amine terminated, and polypeptides as synthetic polymers and alginate, hyaluronic acid, collagen, chitosan, gelatin, dextran and silk as natural polymers (Table 1). Hydrogels can be created by chemical and physical crosslinking. One may readily produce hydrogels using physical mechanisms such as heat and ionic gelation, self-assembly, and electrostatic interactions. However, their qualities mostly rely on the inherent characteristics of the polymers and the hydrogels made using these procedures have a limited tolerance for fine-tuning. Contrarily, chemical crosslinking techniques enable more precise crosslinking but typically call for the alteration of the polymers, which may compromise their biofunctionality [82,83,84,85,86].

Physical crosslinking can be achieved by different methods. These methods include thermo condensation (Figure 1B), self-assembly (Figure 1C), ionic gelation (Figure 1D) and electrostatic interaction (Figure 1E). Thermo condensation happens when the polymer chains get physically entangled as a result of temperature fluctuations during the gelation process. Self-assembly depends on noncovalent bonds like hydrogen bonds, van der Waals forces, and hydrophobic interactions. Ionic and electrostatic interactions are caused by the interaction of the opposing charges. Chemical crosslinking (Figure 1F), on the other hand, is achieved by covalent bonding of chemically active polymers. Numerous techniques have been employed, including radical polymerization, aldehyde complementation, high-energy irradiation, click chemistry, carbodiimide chemistry, enzyme-enabled biochemistry and azide-alkyne reactions. When compared to physical processes, chemical crosslinking techniques result in greater stability in the hydrogel matrix. They also provide more control and flexibility of hydrogel production.

**Figure 2:**
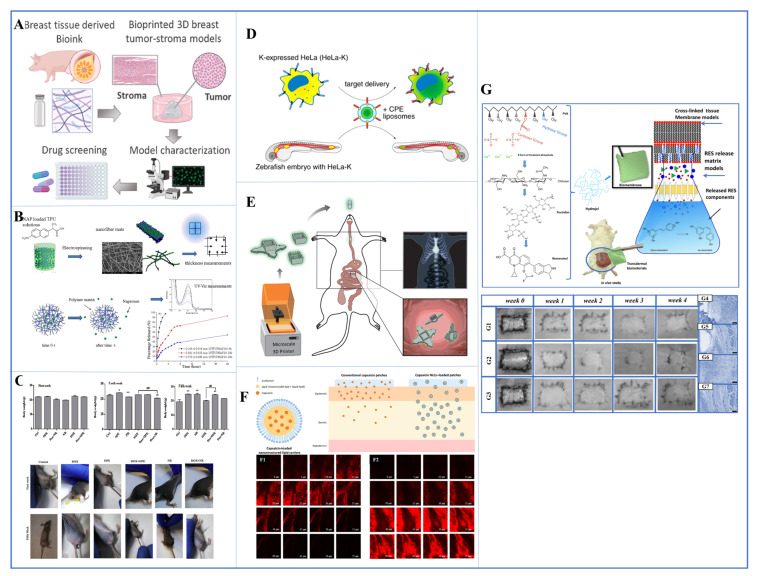
(**A**) 3D bioprinted breast tumor-stroma tumor models Reproduced with permission from [93], Elsevier, 2023. (**B**) naproxen-loaded thermoplastic polymer drug delivery system with electrospinning method Reproduced with permission from [102], Elsevier, 2016. (**C**) the antitumor effect of Dox combined with OPE and NR in vivo (xenograft tumor mouse model) Reproduced from [112], Elsevier, 2020. (**D**) zebrafish xenograft model Reproduced from [113], ACS publication, 2016 (**E**) schematic illustration of the 3D-printed microcontainers (Eudragit L100 and furosemide) that were appropriate as drug carriers Reproduced with permission from [114], Wiley Online library, 2023. (**F**) graphical abstract of transdermal patches embedded in nanostructured lipid carriers (NLCs) to improve transdermal delivery of capsaicin Reproduced with permission from [115], Elsevier, 2022. (**G**) in the in vivo wound model and in vivo persistence and histology images of resveratrol-loaded transdermal biomembrane Reproduced with permission from [116], Elsevier, 2023.

Collagen that has been partly hydrolyzed into gelatin may be treated and altered with ease utilizing various techniques and chemistries. Hydrogels are created by the heat condensation of gelatin taken from a variety of animal sources, a method which has been widely researched. Gelatin can also be modified with methacryloyl residues, which could be a good example of the chemical crosslinking of gelatin to produce materials [87,88,89,90]. The produced hydrogels were examined as prospective instruments for delivery of drugs and genes, as well as for the regeneration of various tissues. Through the use of enzymes, decellularized tissue extracellular matrix (dECM) may be converted into biomimetic hydrogels by being neutralized to temperature and pH. They may be extracted from any tissue, and depending on the source tissue and decellularization method, they produce hydrogels with distinct biochemical, architectural, and viscoelastic properties. This led to the creation of several dECM bioinks (Figure 2A) from a variety of organs and tissues. The use of dECM bioinks to control stem cell differentiation and produce biomimetic and functional tissues has enormous promise for use in disease modeling, drug monitoring and regenerative medicine [91,92,93].

The rate at which pharmaceuticals are released from DDSs can be regulated by factors such as the mesh size of the hydrogel network, degradation rate, and swelling ratio. The majority of these characteristics can be changed by varying concentration and crosslinking intensity. This allows them to be specifically engineered to deliver a variety of bioactive molecules [82,84]. Hydrogels can be made to respond to changes in their surroundings or other stimuli. However, cross-linked networks may hinder enzyme penetration, slowing down drug release and degradation. Developments in material science include hydrogels that are capable of repairing themselves after taking damage. The hydrogel’s structure and electrostatic forces stimulate the development of new bonds by non-covalent hydrogen bonding or reconstructive covalent dangling side chains. These characteristics, which resemble flesh, have encouraged research and development on self-healing hydrogels for use as scaffolds in passive and preventative applications as well as in reconstructive tissue engineering [94,95,96].

**Table 1:** Components used in polymeric-based, drug-loaded hydrogel drug delivery systems, applications, methodology and advantages.

Biocomposite			Applications	Methodology of Produced Hydrogel	Advantages	Ref.
Component 1	Component 2	Component 3				
PVA(Polyvinyl Alcohol)	Alginate	Rosuvastatin (RSV) (Drug)-loaded Chitosan	Elimination of overdose, improves solubility of drugs	Solvent Casting Method	Elimination of overdose, improves solubility of drugs(PVA), Rate of drug release can be controlled by using different layers in hydrogels, nontoxicity, biodegradability, immunogenicity, biocompatilibilty.	[97]
PLA—Poly(Lactic)	PEG(Polyethylene Glycol)	Curcumin(Drug)	Wound dressing	Electrospinning	PLA increases the bioavailability and performance of curcumin in aqueous media, poly(ethylene glycol) (PEG) is widely used in pharmaceutical formulations due to its ability to increase the aqueous solubility of poorly soluble substances. This is owing to its specific properties, which include non-toxicity, great biocompatibility, and easy clearance from the human body. These qualities make PEG an important component in medicines, allowing the solubilization of otherwise weakly soluble chemicals, hence increasing their efficacy and bioavailability.	[98]
PNIPAAm	Curcumin(Drug)	-	Anti-cancer drug delivery	Free Radical Polymerization	PNIPAAM provides both pH- and temperature-sensitive drug release. It is biocompatible, and the loaded drug has the capacity to be released in response to the intracellular microenvironment.	[99]
Collagen	Recombinant Rat Nerve Growth Factor Beta (NGF-β)	-	Neuroregenerative Drug Delivery	Compression Molding	Favorable optical and mechanical properties to be applied in corneal stroma, collagen provided migration of host stromal cells	[100]
Chitosan Hydrogel	Graphene Oxide	Teriparatide	Regeneration of osteoporotic bone defects by utilizing photothermal responsive graphene oxide modified chitosan hydrogel	Electrodeposition	Reduced graphene oxide provides better photothermal conversion property. Also, this property enhanced the release of drug molecules	[101]

### 2.3. Thermoplastic Drug Delivery Systems

When creating DDSs, thermoplastics provide a variety of benefits over other polymers [102,103,104]. They offer flexibility to modify mechanical characteristics and degradation to be used in different applications, and the ability to construct synthetic polymers into a variety of geometries using a variety of production techniques like casting, electrospinning, and 3D printing. Poly-(lactic acid) (PLA), poly(lactic-co-glycolic acid) (PLGA) and polycaprolactone (PCL) are some of the common thermoplastic polymers used in DDSs. These polymers are commonly hydrophobic and used for delivering hydrophobic drugs most of the time. One of their main benefits is maintaining stability while not requiring any crosslinking steps [105,106]. By changing PLA’s stereochemistry, one can change its thermal, mechanical and biological characteristics. Furthermore, by varying the proportion of PLA and PGA in the copolymer, further biodegradability in PLGA can be adjusted. Though local acidity increases resulting from decomposition may cause problems at the application site, PLGA is usually considered the “gold standard” among biodegradable polymers. According to in vitro release tests, the ratio of PLGA/PLA affected the rate at which DOX was released, with a higher PLA content. Additionally, it was shown that varying PEG concentrations can affect the release of DOX, since a rise in PEG corresponds to a marked rise in the rate of DOX release [107]. To create drug delivery systems, thermoplastic polymers can be used with a variety of fabrication processes. For instance, docetaxel was added to the poly(D,L-lactide) nanofibers by Ding et al. It was discovered that DTX may be released continuously from nanofibrous membranes in vitro for 24 days this way. In studies conducted on mice, it was observed that mice treated with drug-loaded membranes experienced a significantly lower rate of locoregional recurrence after the removal of the primary tumor compared to mice treated with systemically administered DTX or locally administered DTX. This suggests that drug-loaded membranes may be more effective in preventing local tumor recurrence in this experimental model. The absence of inflammation in the tissue surrounding these drug-loaded membranes underlined their high level of biocompatibility. In a distinct research study, nanofibrous membranes constructed from poly(L-lactic acid) and coated with 5-fluorouracil were designed to combat colorectal cancer in xenografted mice. The in vivo experiments revealed that these membranes were more effective at preventing tumor growth compared to intraperitoneal injection of 5-FU. This improved efficacy was attributed to the sustained and continuous release of 5-FU from the membranes, providing a more targeted and controlled approach to combating colorectal cancer [108]. In recent developments, 3D printing technology has been employed to fabricate implantable drug delivery systems using thermoplastic biopolymers. A recent innovative example in drug delivery involves the extrusion printing of a 3D patch made from a combination of PLGA and PCL, which is loaded with 5-fluorouracil. This 3D-printed patch is designed for the prevention of pancreatic cancer growth, showcasing the potential for tailored drug delivery systems specifically designed to address medical applications like cancer treatment. This approach combines advanced materials and 3D printing technology to develop more precise and effective drug delivery solutions [109,110]. In a different work, developed materials made of electrospun nanofibers that are loaded with drugs. Thermoplastic polyurethane (TPU) solutions at 8 and 10% (w/w) concentrations were electrospun to produce TPU ultrafine fiber mats that contain naproxen (NAP). The solutions comprised 10 and 20 percent NAP, respectively, based on the weight of TPU. The drug-loaded electrospun TPU fibers were collected at 5, 10, and 20 hours, and their properties were evaluated using Fourier transform infrared spectroscopy, differential scanning calorimetry, and thermogravimetric analysis. Average diameters of the NAP-loaded electrospun TPU fiber mats ranged from 523.66 to 723.50 nm, and they exhibited a smooth shape. To analyze the release characteristics of these fiber mats, the entire immersion method in a phosphate buffer solution at 37 °C was employed. It was observed that the duration of mat collection significantly influenced the release of NAP (Figure 2B) [102]. In a similar study, blend formulations for drug-eluting implants designed for prolonged loading have been developed. These formulations combine a high-modulus thermoplastic segmented polyurethane (TSPU) with poly(L-lactide) (PLLA). The production of various blend compositions was achieved by solution casting. This process is part of the development of drug-eluting implant materials for medical applications. Both strip- and tube-shaped samples underwent an elastic recoil test, and the tensile elastic recovery (TER) and relative elastic recovery (RER) were calculated. To assess the mechanical properties of various blend compositions and pure polymers, a uniaxial tensile test was performed [111]. In conclusion, by creating blends with various PLLA/TSPU ratios, a wide variety of DDSs with diverse extendibility, expandability, and elasticity can be created. An important benefit of using these polymer blends as implantable DDSs is the absence of the need for any plasticizer or compatibilizer.

### 2.4. Microdevice Delivery Systems

Recent progress in the field of drug delivery has led to the development of integrated micro drug delivery devices [117,118,119,120,121]. These systems combine device technology with therapeutic molecules to create implanted devices that can provide disease therapy directly at the treatment site. Depending on their specific design, these microdevices offer various options, such as continuous or intermittent drug administration, and can function effectively for both short-term and long-term treatment regimens. This represents a significant step forward in the precision and effectiveness of drug delivery for various medical conditions. Similar to conventional drug delivery systems, these gadgets can either passively supply a medication or enable real-time drug dose changes through an outside stimulation. Jonas and his colleagues developed a tiny cylindrical device that uses micromachining to discharge a variety of medications using 16 distinct reservoirs around its periphery. The gadget was promptly put into tumors of various sorts utilizing a biopsy procedure, and it was left in place for 24 hours in order to quickly and simultaneously examine drug sensitivity in in vivo tumors. A coring biopsy needle was then used to remove a small portion of tissue from the device in order to assess each treatment’s potential anti-cancer effects. Three distinct methods were employed to alter the release rates of each medication in the reservoir. As a result, it was demonstrated that the device could administer a single drug or a combination of treatments into limited areas of cancer tissue. The researchers also assessed the pharmacokinetics of DOX release in breast cancer tumor xenografts (Figure 2C) and found that the medication delivered by the device can spread 200 to 300 nm into cancer tissue [112,113,122]. In another study, a significant effort was made by using photolithography to construct a temozolomide (TMZ)-based Microelectromechanical System Sensor (MEMS) microdevice that featured moving elements that could be controlled by an external magnet and was biocompatible and biodegradable. A mouse model was used to test in vivo the single-gear and Geneva drive variants of this device. A multireservoir gear was part of the single-gear system; one of the reservoirs contained iron oxide nanoparticles, while the others contained pharmaceuticals. Contrarily, the Geneva drive has two connected gears: a driven gear with six drug-filled chambers and a driving gear laced with iron oxide nanoparticles. The medicine was released from each reservoir when the top opening lined up with another opening on the uppermost part of the manufactured device, which was the same mechanism for both variants. An external magnet moved the gears, which turned them. Successful in vivo gear movement and controlled dye release were observed when the Geneva drive device was loaded with two different fluorescent dyes and subcutaneously implanted in a mouse model [123]. Another study involved a zebrafish xenograft (Figure 2D) that was injected next to E4-Lipo-TP3 or E4-Lipo-DOX into zebrafish xenografts of HeLa-K. As a result, E4-liposomes delivered TP3 to the implanted HeLa-K cells, and E4-Lipo-DOX could suppress cancer proliferation in the xenograft when compared to nontargeted conditions (i.e., zebrafish xenograft with free DOX injection). This resulted in enhanced cytotoxicity toward HeLa-K cells compared to free doxorubicin [113]. Another study described a technique for creating radiopaque microdevices with enhanced mucoadhesive geometries for testing and 3D printing. Three distinct microcontainer designs were developed and manufactured with the goal of extending gastrointestinal retention and managing the direction of one-way drug release. In order to determine if the 3D-printed microcontainers were appropriate as drug carriers for oral delivery, they were filled with the small molecule medication furosemide and coated with the pH-sensitive polymer Eudragit L100. The goal of the current work was to demonstrate that 3D-printed microcontainers can be used for oral medication delivery in a manner that is consistent with other types of microcontainers that have been previously reported. An Eudragit L100 coating was selected because of its suitable pH sensitivity and lack of enzymatic activity; it is commonly employed for oral medication administration to the small intestine. For an in vivo rat study, a comprehensive experimental design was developed, involving 24 rats. The study involved a 30-minute gastric phase, succeeded by an intestinal phase, lasting until complete furosemide [124] release was achieved. This release pattern resembled that of microcontainers produced using previous methods. Therefore, the 3D-printed microcontainers displayed promising results as an oral drug delivery system, particularly for targeted distribution to the small intestine when combined with a pH-sensitive polymeric coating (Figure 2E) [114].

### 2.5. Transdermal Patch Delivery Systems

Transdermal DDSs are designed to stick to the surface of the skin in order to permit the transfer of the active chemicals through the skin layers for either a local or systemic effect. Transdermal sprays, lotions, patches, and implantable devices can all be used to accomplish this. For transdermal patches, the medication is frequently housed in a reservoir with a porous membrane around it. Alternatively, it might be enclosed in an adhesive matrix that melts at body temperature to release the medication embedded therein [125,126,127,128,129]. In previous work, a transdermal DDS was developed for the first time to deliver prophylactic antibiotics. Solid lipid nanoparticles (SLNs) loaded with the vitamin E-based antibiotic cephalexin were created into a pressure-sensitive transdermal patch. After loading into TOS-based SLNs with different drug-to-lipid ratios, cephalexin was dispersed in PIB-based adhesive solutions using a weight percent ratio of 60:40 with PIB-B10 and PIB-B50. The incorporation of nanoparticles also resulted in an approximately twofold increase in drug loading. By using this optimal formulation, it is possible to reduce drug use by approximately 28% while maintaining antibacterial efficacy. In vitro drug release, antimicrobial activity and skin cell proliferation properties of transdermal patches were evaluated. It has been observed that the growth of human fibroblast skin cells in optimal patch-containing media occurs at approximately 25.5% proliferation [130]. In similar studies, they developed transdermal patches containing capsaicin nanostructured lipid carriers (NLCs), a drug used to treat skeletal muscle and neuropathic pain. These capsaicin-loaded NLCs were then incorporated into polyacrylic polymers to create transdermal patches with a consistent capsaicin concentration of 0.025 percent. In vivo studies showed that traditional capsaicin drug-loaded patches can cause skin irritation and redness, while capsaicin NLC-loaded patches exhibited lower skin side effects. Therefore, capsaicin NLC-loaded patches have been shown to be a potential transdermal delivery system for capsaicin, possibly reducing skin irritation (Figure 2F) [115]. In other similar studies, the design and production of transdermal membranes loaded with resveratrol and added with fucoidan and chitosan bioactive substances based on polyvinyl alcohol/β-tricalcium phosphate were carried out. In addition to in vitro biocompatibility (MTT test) and in vivo studies on resveratrol-loaded transdermal membranes, bioadhesion strength was also calculated. The shelf life of resveratrol-loaded transdermal membranes was also calculated using the Minitab program. In vivo wound healing in 4-week-old mice was estimated to be 98.75%, while the shelf life of resveratrol on transdermal membrane was estimated to be approximately 36 days. This study demonstrated that the innovative and novel transdermal biomaterial promoted tissue cell regeneration and cell proliferation in theranostic applications as wound dressings (Figure 2G) [116].

## 3. Nanobiotechnology Drug Delivery Systems

Materials at the nanoscale are employed to administer therapeutic drugs or as diagnostic instruments in nanomedicine, a field of study that is still relatively young but evolving quickly [131,132]. Nanomaterials can mimic the endocytosis mechanism used by viruses to get through a cell membrane [133]. To achieve their objectives, nanomaterials must possess specific characteristics. Each particle must circumvent two elimination systems that threaten its circulation time: renal clearance and phagocytosis. Therefore, it’s crucial to fine-tune the size and shape of these particles to optimize their half-life in circulation and their ability to enter cells. To prevent renal clearance, these particles should have a size greater than 10 nm, while to evade phagocytosis, their size should be smaller than 500 nm. This size range ensures they can effectively navigate both elimination systems [134].

### 3.1. Nanoparticle Internalization and Transport Mechanisms in Drug Delivery Systems

Before delving into the various nanomaterials utilized in nanobiotechnology-based drug delivery systems, it is essential to understand the mechanisms of nanoparticle internalization and transport. These processes, including endocytosis, clathrin-mediated endocytosis [135,136,137], caveolae-mediated endocytosis [138,139,140], and micropinocytosis [141,142,143], play a pivotal role in determining the efficiency and specificity of nanoparticle-mediated drug delivery. A thorough understanding of these mechanisms enables the rational design of nanocarriers capable of effectively targeting specific cells or organs while minimizing off-target effects.

Endocytosis represents the primary cellular mechanism for the internalization of nanoparticles and other extracellular substances [144,145]. This process involves the engulfment of particles through the formation of vesicles derived from the plasma membrane. Endocytosis is broadly categorized into phagocytosis and pinocytosis, with the latter being more relevant for nanoparticle uptake in non-phagocytic cells. Pinocytosis is further divided into clathrin-mediated endocytosis, caveolae-mediated endocytosis, and macropinocytosis, each characterized by distinct cellular pathways and mechanisms.

#### 3.1.1. Clathrin-Mediated Endocytosis

Clathrin-mediated endocytosis is one of the most extensively studied nanoparticle internalization mechanisms [135,137]. This process involves the formation of clathrin-coated pits on the plasma membrane, which invaginate to form vesicles. These vesicles transport nanoparticles to early endosomes and subsequently to lysosomes for further processing or degradation. The size, charge, and surface functionality of nanoparticles are critical factors influencing their uptake via this pathway. Nanoparticles functionalized with ligands targeting clathrin-associated receptors, such as transferrin receptors, exhibit significantly enhanced internalization efficiency [146,147,148].

#### 3.1.2. Caveolae-Mediated Endocytosis

Caveolae-mediated endocytosis involves small, flask-shaped invaginations in the plasma membrane enriched with cholesterol, sphingolipids, and caveolin proteins [139,149]. Unlike clathrin-mediated pathways, caveolae-mediated endocytosis often bypasses lysosomal degradation, making it an advantageous route for the delivery of sensitive therapeutic agents such as proteins or nucleic acids [150,151,152]. Nanoparticles designed to exploit this pathway typically feature hydrophobic or amphiphilic surface modifications that mimic the lipid-rich environment of caveolae, enhancing their interaction and uptake.

#### 3.1.3. Macropinocytosis

Macropinocytosis is a non-specific internalization mechanism characterized by the formation of large vesicles known as macropinosomes [141,153,154,155]. This process is often induced by cellular signaling events that trigger membrane ruffling, leading to the engulfment of extracellular fluid and particles. While macropinocytosis lacks the specificity of receptor-mediated pathways, it provides a high-capacity route for the uptake of larger nanoparticles or aggregates. Surface properties, such as hydrophilicity and charge, can significantly influence the efficiency of macropinocytic uptake [156,157,158].

#### 3.1.4. Implications for Nanocarrier Design

The choice of internalization pathway has profound implications for nanocarrier design. For instance, nanoparticles targeting clathrin-mediated endocytosis necessitate precise ligand-receptor interactions, while those leveraging caveolae-mediated pathways benefit from structural mimicry of lipid rafts [159,160,161]. Furthermore, the size and morphology of nanoparticles must be optimized to facilitate interactions with specific endocytic pathways. Particles in the 10–100 nm range are generally ideal for clathrin-mediated endocytosis, whereas larger particles are more likely to engage macropinocytosis. Morphological features, such as spherical, rod-like, or platelet shapes, further influence the efficiency of cellular uptake and the selection of internalization pathways.

In conclusion, a comprehensive understanding of nanoparticle internalization and transport mechanisms is fundamental to advancing nanobiotechnology-based drug delivery systems. By leveraging these pathways, researchers can design nanocarriers with enhanced specificity, efficiency, and therapeutic potential. Such knowledge allows for the rational development of tailored nanomaterials for specific biological applications, ultimately improving the efficacy and precision of modern therapeutics.

### 3.2. Carbon Nanotubes for Targeted Drug Delivery

A carbon nanotube (CNT) is a structure that looks like a tube formed by rolling a graphene sheet. Its length and diameter are roughly a few hundred micrometers and a few nanometers, respectively. The primary advantages of CNTs are their high surface area for transporting several medications, sp2-hybridized structure, which may draw drug molecules and targeting agents, and ability to penetrate cells [162,163,164,165].

**Figure 3:**
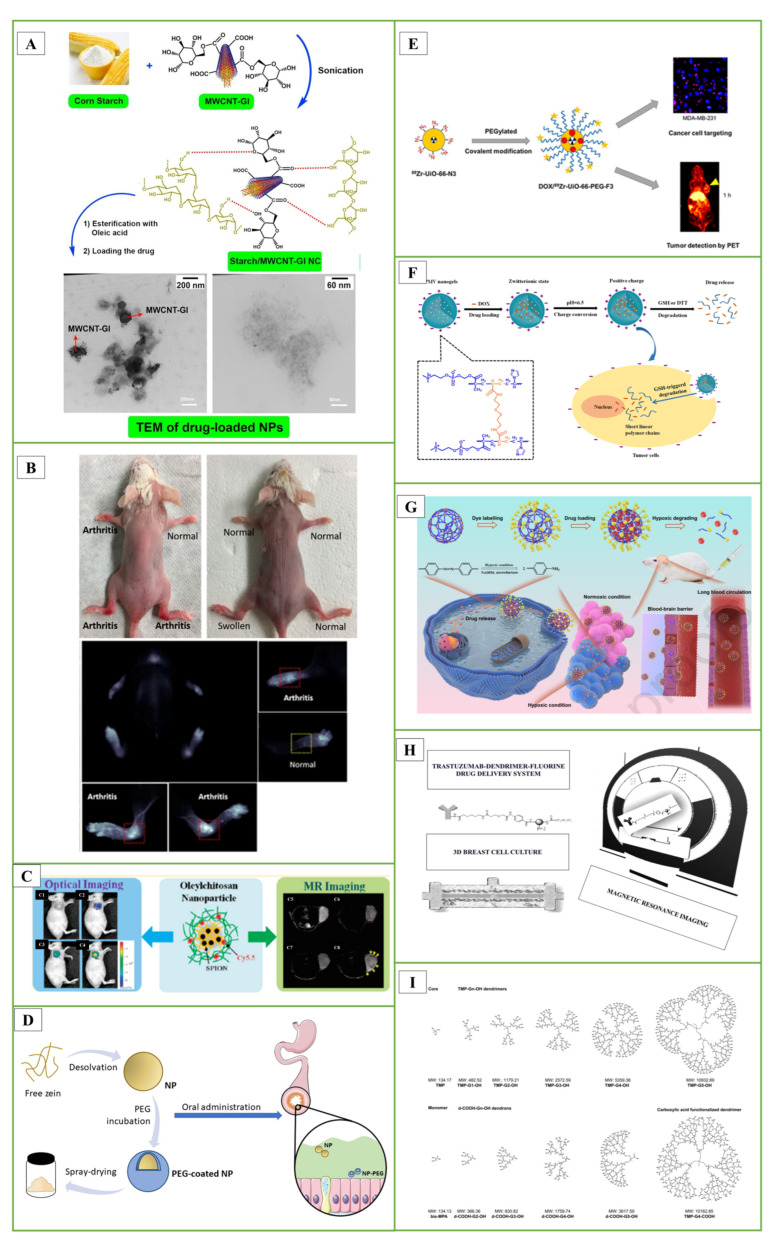
(**A**) The sonochemical method was used to prepare starch/MWCNT-Gl NCs nanoparticles for drug delivery Reproduced with permission from [166], Elsevier, 2018. (**B**) Arthritis imaging of HiPco-cy5.5 in the control group and mice with highlighted arthritis and normal joint Reproduced [167], MDPI, 2021. (**C**) Oleic acid-conjugated chitosan (oleyl-chitosan) is a powerful platform for encapsulating oleic acid-decorated iron oxide nanoparticles (ION) Reproduced with permission from [178], ACS Publications, 2018. (**D**) Graphical abstract of PEG-coated Zein nanoparticles Reproduced [183], Elsevier, 2021. (**E**) Graphical abstract of DOX/Zr-UİO-66-PEG-F3 Reproduced with permission from [184], ACS Publications, 2021. (**F**) DOX-loaded phosphorylcholine-based zwitterionic polymer nanogels’ charge-conversion ability at tumor extracellular pH Reproduced with permission from [201], Elsevier, 2019. (**G**) Schematic illustration of the poly(phosphorylcholine)-based (HPMPC) nanogel with long blood circulation, blood-brain barrier (BBB) penetration, and hypoxic controlled drug release for glioblastoma drug delivery Reproduced with permission from [202], Elsevier, 2021. (**H**) Trastuzumab-dendrimer-fluorine drug delivery system’s efficacy can be evaluated in 3D breast cell culture Reproduced from [203], Elsevier, 2021. (**I**) Bis-MPA dendrimers and related structures Reproduced with permission from [204], Elsevier, 2012.

In the studies, starch nanocomposite (NC) films containing carbon nanotubes (MWCNTs) were designed. To increase the hydrophilicity of MWCNTs, its surface was modified with the D-glucose (Gl) biomolecule. Starch was loaded onto MWCNT-G1 NC by sonochemical method. Then, pure starch and starch/MWCNT-Gl NCs were reacted with oleic acid to obtain amphiphilic (Amph) esters. The hydrophobic anti-insomnia drug zolpidem was loaded onto NCs for drug delivery. The entrapment efficiency, loading capacity and in vitro release study of zolpidem as a hydrophobic drug model were performed (Figure 3A) [166].

In another study, they evaluated single-walled CNTs, specifically HiPco-SWCNT and carboxyl-SWCNT, as carriers for delivery of an anti-inflammatory drug, methotrexate, and siRNA targeting the NOTCH1 gene. Methotrexate (MTX) is frequently used to treat rheumatoid arthritis (RA). However, MTX is toxic, and a large proportion of patients receiving MTX alone or in combination with other medications experienced gastrointestinal side effects and hepatotoxicity. HiPco-SWCNTs or carboxyl-SWCNTs were combined with a NOTCH1 siRNA and/or MTX to create 12 different nanotube products. The products were then subjected to many tests to see if they could be used as a drug delivery mechanism to reduce the side effects of MTX. According to in vivo studies, HiPco-SWCNTs assemble in arthritic joints. The %CE of MTX for HiPco-SWCNTs was between 77% and 79%; for siRNA binding efficiency, this rate was up to 97%. SWCNTs showed dose-dependent interactions with monocytes, neutrophils, and to a lesser extent B cells when cultured with human blood. Notably, the incorporation of siRNA increased the adsorption efficiency of HiPco-SWCNTs. These findings indicate that HiPco-SWCNTs are effective drug delivery systems that can be used in the treatment of RA (Figure 3B) [167].

### 3.3. Nanoparticle Drug Delivery

Nanoparticles are matrix systems prepared with natural or synthetic polymers, ranging in size from 10–1000 nm, called nanospheres or nanocapsules depending on the preparation method, in which the active substance is dissolved in the particle, entrapped and/or adsorbed or bound to the surface. Nanocapsules are vesicular systems, in which the drug is entrapped in a cavity and surrounded by a polymer membrane. Nanospheres are matrix systems where the drug is physically and uniformly dispersed [8,131]. The advantages of nanoparticles, which are obtained by using natural or synthetic polymers and used for targeting drugs as well as proteins, peptides and genes to the relevant tissue, stem from two main features. The first is that nanoparticles have small particle sizes. Thus, they are taken into the cells by passing through small capillaries and provide accumulation of active substance in the target area. The second is the use of biodegradable materials in the preparation of nanoparticles. Biodegradable materials provide controlled release of active substances in the target tissue over periods of days or even weeks. In addition to all these, nanoparticles increase the stability of drugs, proteins or peptides; can be easily sterilized; have a high active substance loading capacity; and thus increase the intracellular distribution of the active substance. In this way, the release and bioavailability of the drug given as nanoparticles in oral drug administration increases [131].

Advanced synthesis techniques have made it possible to use a variety of materials and produce a large range of nanoparticles in different sizes and shapes. Nanoparticles can be categorized based on various physical and chemical attributes. Different types of nanostructures, including metalloid, organic, inorganic, and polymeric ones like dendrimers, micelles, and liposomes, are commonly considered when designing drug delivery systems for specific targets. These nanoparticles are particularly useful for enhancing the delivery of drugs with poor absorption and limited solubility. However, the effectiveness of these nanostructures as drug carriers can vary significantly based on factors such as their size, shape, and other inherent biophysical and chemical properties. Polymeric nanoparticles with a diameter of 10 to 1000 nm have characteristics that make them superior delivery devices.

A wide array of synthetic polymers, such as polyvinyl alcohol, poly-L-lactic acid, polyethylene glycol, and poly(lactic-co-glycolic acid), as well as natural polymers like alginate and chitosan, are extensively employed in the fabrication of nanoparticles [168,169,170,171]. Their use is driven by their excellent biocompatibility and biodegradability. These polymeric nanoparticles, both nanospheres and nanocapsules, offer effective drug delivery methods. Additionally, lipid-based nanostructures, including compact lipid nanoparticles and phospholipids, are highly valuable for targeted drug delivery, much like liposomes and micelles [172,173,174].

#### 3.3.1. Coated Nanoparticles

By coating nanoparticles with surfactants or physiological elements like serum complement factors, one can change the dispersion of the particles within the body. To target binding drugs to tumors, the brain, and inflammatory bodily regions, different coated nanoparticles can be employed [175,176,177].

In previous studies, they encapsulated oleic acid-coated FeO nanoparticles in oleic acid-conjugated chitosan (oleyl-chitosan) to investigate the uptake of nanoparticles by tumor cells. They aimed to study the in vivo penetration and retention of these nanoparticles for analytical applications using near-infrared and magnetic resonance imaging techniques. After intravenous administration of cyanine-5-linked oleyl-chitosan nanoparticles, both imaging modalities exhibited a noticeable increase and improvement in signal intensity in tumor tissues, primarily due to the increased EPR effect. This research demonstrated the potential utility of these nanoparticles for imaging and analytical purposes (Figure 3C) [178].

In another study, a biomimetic nanoparticle formulation designed to target inflammation by exploiting the specific interaction between very late antigen-4 (VLA-4) and VCAM-1 was developed. In this approach, polymeric nanoparticle cores are surrounded by plasma membrane from cells genetically modified to consistently express VLA-4. Consequently, these cell membrane-coated nanoparticles exhibit enhanced affinity towards target cells overexpressing VCAM-1, as demonstrated in vitro. Additionally, this formulation involves the encapsulation of dexamethasone, a well-known anti-inflammatory drug. This improves drug delivery to inflamed lung tissues and greatly increases its therapeutic efficacy in vivo. The overall goal of this research is to exploit naturally occurring target-ligand interactions and take advantage of the different properties of biological membrane coatings to develop nanoparticles with greater targeting specificity [179].

#### 3.3.2. Pegylated Nanoparticles

The target molecule is commonly PEGylated by being incubated with a reactive PEG derivative [180,181]. By increasing the agent’s size in solution and lengthening its circulation duration by reducing renal clearance, covalent attachment of PEG to a medication or therapeutic protein may “hide” the agent from the host’s immune system, reducing immunogenicity and antigenicity while increasing its size in solution. PEGylation is able to make hydrophobic proteins and drugs soluble in water. PEGylation technology is expanding quickly and has proven to have therapeutic effects [182].

In a study, PEG-coated nanoparticles (NP-PEG) with mucus-permeable properties were produced for oral drug delivery. For this purpose, zein nanoparticles were prepared by desolvation and then coated by incubation with PEG 35,000. The hydrophobic surface of zein nanoparticles (NP) was significantly reduced due to their coating with PEG. This increase in the hydrophilicity of PEG-coated nanoparticles was associated with a significant increase in their mobility in porcine intestinal mucus. After oral administration, NP appeared to remain trapped in the mucus network, whereas NP-PEG was able to penetrate the protective mucus layer and reach the epithelium. It was concluded that PEG-coated zein nanoparticles could be adequate carriers to promote the oral bioavailability of biomacromolecules and other biologically active compounds with low permeability properties (Figure 3D) [183].

In another important study, a zirconium-based DOX UiO-66-N3 was developed, and its surface was covalently functionalized with PEG containing alkynes using azide-alkyne click chemistry. The F3 peptide was then incorporated to specifically target cancer cells. Positron-emitting zirconium-89 was added to UiO-66-N3 to monitor pharmacokinetic behavior in vivo. A corresponding PEGylated F3 peptide was conjugated to provide 89Zr-UiO-66-PEG-F3. Serial PET imaging demonstrated that 89Zr-UiO-66-PEG-F3 accumulated preferentially in MDA-MB-231 tumors and was cleared from the liver more quickly than PEGylated UiO-66 using non-covalent techniques (Figure 3E) [184].

Advantages of PEG-Activity Conjugates

1. PEG masks the protein surface by steric hindrance and can be used to protect against renal damage;
2. It increases the molecular size of the polypeptide, and as a result, renal ultrafiltration is reduced;
3. The contact of the antibody or antigen processing cells with PEG chains is also inhibited;
4. Protein immunogenicity is reduced or eliminated;
5. PEG carries its physicochemical properties to the peptide or nonpeptide molecule to which it binds, and thus the bioavailability and solubility properties of the substance are altered;
6. Enzymes and bioactive substances dissolve in organic solvents or aqueous solutions;
7. In vivo, the excretion of PEG-protein conjugate and its circulation time in the blood are prolonged;
8. Stabilizes the physiological properties of proteins and bioactive substances;
9. Improves the pharmacokinetic properties of various active substances;
10. Increases accumulation in tumor tissues [19].

#### 3.3.3. Solid Lipid Nanoparticles (SLN)

Polymer-based nanocarriers offer various advantages due to their versatility in enabling a wide range of modifications, like the creation of block and comb polymers. Solid lipid nanoparticles represent one such type of nanocarrier design that capitalizes on the strengths of colloidal carriers while circumventing their limitations [185,186,187]. SLNs typically consist of approximately 0.1–30% (*w*/*w*) solid fat and exhibit an average size ranging from 150 to 300 nm, although their size can extend up to 1000 nm based on the surfactant employed during the manufacturing process. The behavior of SLNs is influenced by their size and the ratio of solid to liquid fat. Some advantages of SLNs include the ability to load both lipophilic and hydrophilic therapeutic agents, high drug loading capacity, easy synthesis in larger quantities, and minimal to no adverse effects on healthy tissue. One of the most common applications of SLNs as nanocarriers is for the oral administration of medications [187,188]. In the study, they developed solid lipid nanoparticles through a heated, high-pressure homogenization process. They conducted a study to evaluate how independent variables, specifically surfactant and lipid ratio, affect the physicochemical properties of SLN, including average particle size (Z-Ave), polydispersity index (PDI), and zeta potential (ZP). ZP measurements were obtained using electrophoretic light scattering, while Z-Ave and PDI were determined using dynamic light scattering. Findings show that the optimal SLN dispersion contains 1 weight percent α-pinene, 4 weight percent solid lipid, and 2.5 weight percent surfactant, resulting in Z-Ave at 136.7 nm, PDI at 0.170, and 0 mV ZP. Additionally, the stability analysis of α-pinene loaded SLN was effectively carried out using LUMISizer^®^ (Berlin, Germany) [189].

#### 3.3.4. Smart Polymeric Nanogels

Polymeric nanovehicles have demonstrated exceptional capabilities for encapsulating and delivering theranostic substances under physiological conditions. They can even be utilized for monitoring therapeutic response. Presently, polymer nanogels are widely recognized as efficient delivery systems for a diverse range of therapeutic and diagnostic compounds [190,191,192,193,194]. Notably, biodegradable and “intelligent” nanogels made from intelligent polymers offer substantial advantages due to their ability to respond to both endogenous and exogenous stimuli, including pH gradients, bioresponsiveness, photoresponsiveness, temperature, and more. Many multifunctional nanogels with excellent targetability and sensitivity have been developed for various theragnostic applications [193,194,195,196,197,198].

The current synthesis techniques for these nanogels can be broadly categorized into four groups [199,200]: **(i)** homogeneous or heterogeneous monomer polymerization; **(ii)** appropriate polymer self-assembly; **(iii)** suitable polymeric architecture cross-linking; and **(iv)** template-assisted nanogel synthesis. These techniques have found applications in a variety of theragnostic contexts [191]. In the literature study, using a one-step reflux precipitation polymerization process, they formed a series of biodegradable poly(2-methacryloyloxyethyl phosphorylcholine-s-s-vinylimidazole)(PMV) nanogels with uniform spherical shape. These nanogels exhibited specific behaviors in response to pH changes. At physiological pH, the PMV nanogels remained in the zwitterionic state but rapidly switched to a positively charged form at the extracellular pH of tumors. A study focusing on protein stability revealed that DOX-loaded PMV nanogels could resist protein adsorption at pH 7.4 for up to 7 days. However, they readily absorbed proteins at pH 6.5. This behavior highlights the potential of these nanogels for controlled interactions against tumor cells under varying pH conditions (Figure 3F) [201]. In another study, hypoxia degradable zwitterionic phosphorylcholine nanogels called HPMPC were produced as nanoplatforms for chemotherapy treatment of glioblastoma, a malignant tumor. HPMPC nanogels cross-linked with azobenzene were biocompatible and prevented contamination in in vivo studies on mice and showed the ability to circulate in the blood for a long time. Moreover, HPMPC nanogels effectively crossed the blood-brain barriers and provided long-term accumulation in glioblastoma tissue. In conclusion, HPMPC drug nanogels exhibited a positive inhibition effect in the glioblastoma tumor model, making it a potential nanoplatform candidate to treat various hypoxic-related diseases in the central nervous system (Figure 3G) [202].

### 3.4. Delivery Applications of Dendrimers

Dr. Donald Tomalia’s pioneering research on poly(amidoamine) dendrimers was first published in 1985. Dendrimers were first used as a drug delivery mechanism in the late 1990s [205]. Two methods have been used to use dendrimers for drug delivery: formulation and nanostructure. In the nanostructure approach, drugs are covalently bound onto dendrimers, while in the formulation approach, drugs are physically entrapped within a dendrimer using non-covalent interactions [206,207,208,209].

In the studies, we synthesized and characterized various fluorinated dendrimers and designed a Trastuzumab-dendrimer-fluorine drug delivery system for use in breast cancer. A 3D breast cancer cell culture was grown in a bioreactor device. Using MCF-7 cells with Her-2 overexpression in cell culture, the efficacy of the Trastuzumab-dendrimer-fluorine drug delivery system was studied, and magnetic resonance imaging was used to quantify the efficacy. Results showed that the drug delivery system containing trastuzumab, dendrimer and fluorine was more effective than trastuzumab alone. Amino-functionalized polyester dendrimers, known for their biocompatibility and ability to cleave internal esters, have been described to be used for siRNA to cross the blood-brain barrier (Figure 3H) [203].

In research conducted by Patrik and colleagues, they examined dendrimers composed of 2,2-bis(methylol)propionic acid as nonviral vectors for siRNA delivery. In this study, amino-functional bis-MPA dendrimers effectively facilitated gene transfection in human glioblastoma cells and rat glioma cells, resulting in a 20% reduction in the expression of the target protein (Figure 3I) [204].

## 4. Nanoparticulate Systems for Brain Delivery of Drugs

**Figure 4:**
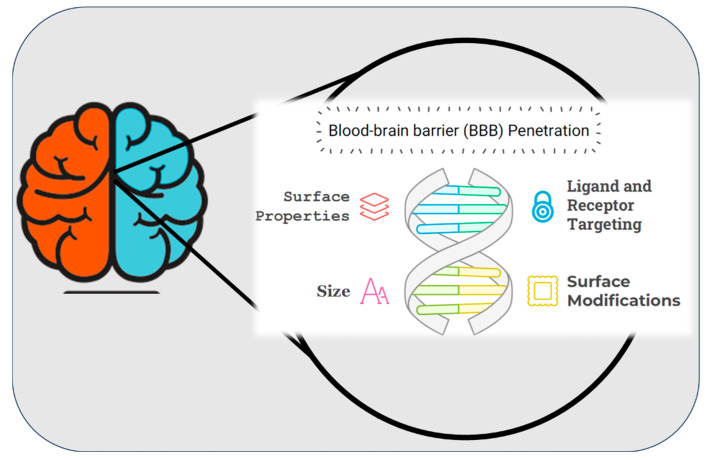
Schematic representation of surface properties, ligand and receptor targeting, and size characteristics of blood brain barrier penetration. “During the preparation of this manuscript, the author(s) used [Napkin AI, beta-0.10.2] for the purposes of [drawing figure]. The authors have reviewed and edited the output and take full responsibility for the content of this publication”.

The development of nanoparticle systems for the delivery of drugs to the brain has emerged as a transformative approach in addressing the limitations associated with conventional drug delivery methods. The primary challenge in treating neurological disorders lies in overcoming the highly selective nature of the blood-brain barrier (BBB) **(**Figure 4), which restricts the passage of most therapeutic agents to the central nervous system (CNS) [210,211,212]. Nanoparticles, owing to their tunable size, surface properties, and biocompatibility, offer a versatile platform to bypass the BBB and enable targeted drug delivery to the brain [213]. Various nanoparticle systems, including polymeric nanoparticles, liposomes, dendrimers, and solid lipid nanoparticles, have demonstrated promising results in preclinical and clinical studies. These systems not only enhance the bioavailability of drugs but also minimize systemic toxicity by providing site-specific delivery [214]. Furthermore, functionalization of nanoparticles with targeting ligands, such as peptides or antibodies, further improves their ability to penetrate the BBB and reach specific neural tissues [215]. This cutting-edge technology has the potential to revolutionize the treatment of neurological disorders, such as Alzheimer’s disease, Parkinson’s disease, and brain tumors, by addressing the unmet need for effective therapeutic delivery. (Neurological disorders and drug distribution methods are shown in Table 2.)

Age-related neurodegenerative diseases (NDs) are very common. Although the central nervous system is the part that is most affected, the peripheral nervous system is also affected. The need to manage or control NDs increased the urge to research and create effective alternative techniques because some medications and diagnostics were unavailable [216,217,218]. Sincere attempts are being undertaken to adapt nanotechnology to manage brain cell activity such as deep brain stimulation, implanted stimulation, therapeutic cargo packing, distribution to the brain, and nanomedicine with improved efficacy. These developments can be used to develop treatment plans in the future that take the patient’s neurodegenerative condition into account. Due to recent developments in targeted delivery, improved effectiveness, and fewer adverse effects of nanotechnology-based techniques, they have been advocated as viable and cheap therapeutic options. Recent studies have been dedicated to utilizing poly(lactide-co-glycolic) acid as a foundational material for the development of therapeutic nanoparticles intended for the treatment of brain tumors and Alzheimer’s disease [219]. In vitro tests have demonstrated that the application of polymeric nanoparticles enhances drug delivery to the brain, reducing oxidative stress, inflammation, and plaque accumulation [220,221,222,223,224,225]. This is achieved through improved curcumin delivery for the treatment of Alzheimer’s disease and efficient internalization of doxorubicin into human glioma cells, resulting in a cytotoxic effect on cancer cells [226,227]. Moreover, in vivo experiments have been conducted involving the simultaneous administration of the anti-cancer drug cisplatin and the antioxidant agent boldine, utilizing poly(lactide-co-glycolic) nanocarriers. These experiments have shown promising targeted delivery for therapeutic applications in brain cancer therapy. In another line of research, the focus has been on andrographolide loaded into nanoparticles based on polyethylcyanoacrylate and human serum albumin [228]. These nanoparticles are designed to address inflammation associated with neurodegenerative disorders.

### 4.1. Blood-Brain Barrier (BBB)

**Figure 5:**
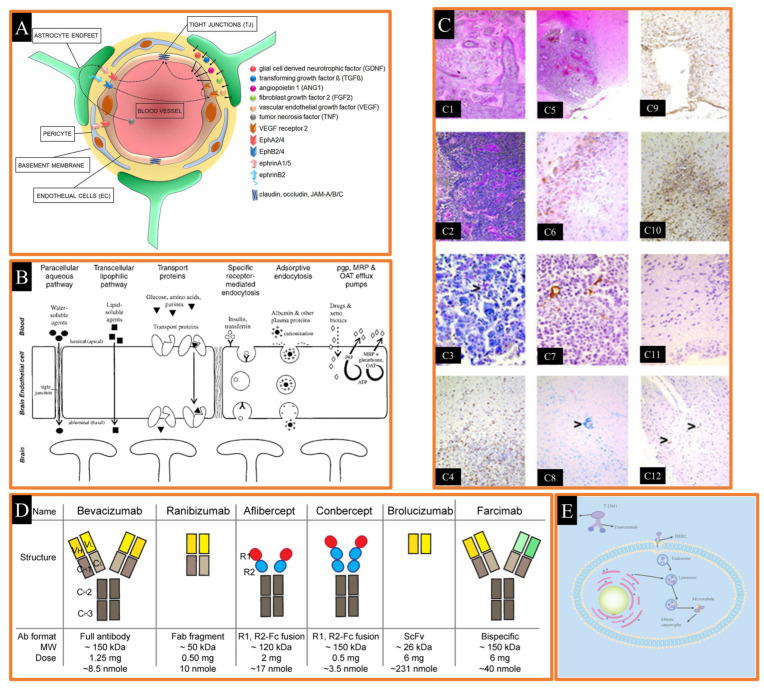
(**A**) Cellular and signaling components of the blood–brain barrier (BBB) in activated EphA and EphB receptors and endothelial cell (EC) junctions (TJ) Reproduced from [232], Frontiers, 2018. (**B**) various crossing mechanisms at the blood brain–barrier Reproduced with permission from [235], Wolters Kluwer, 2004. (**C**) histology of rats with intracranially implanted 101/8 glioblastoma Reproduced with permission from [244], Wiley Online Library, 2004. (**D**) graphical abstract of anti-VEGF antibody-derived drugs Reproduced from [245], MDPI, 2023. (**E**) mechanisms of the action of T-DM1 Reproduced from [243], Frontiers, 2023.

The blood brain barrier (BBB) is created by endothelial cells lining the cerebral microvasculature, and it makes drug targeting to the brain difficult [229,230]. It protects the brain from neurotransmitters and xenobiotics in circulation that might impair neuronal function [210,231]. The BBB is packed with tight connections that block the flow of ions and chemicals. Delivering various drugs to the brain can be challenging due to this reason. Creating a lipid-soluble drug delivery system or utilizing a nanocarrier that can traverse the BBB due to its diminutive size are common noninvasive approaches for administering water-soluble drugs to the brain (Figure 5A) [232].

The BBB is a homeostatic defense mechanism of the brain against pathogens and toxins. It is composed of endothelial cells that form the luminal surface of brain capillaries and are connected by tight junctions. The complex and highly organized BBB maintains the biochemical, physicochemical and structural properties of the substances in its periphery and creates barrier selectivity in the passage of desired molecules into the brain parenchyma. This barrier acts as a selective dynamic filter, preventing the passage of a large number of mostly water-soluble active substances such as antibiotics, antineoplastic agents, peptide-proteins, especially neuropeptides and other oligo- and macro-molecular active substances into the central nervous system [210,233,234]. In order for an active substance to cross the BBB, it must be lipid soluble, non-ionized at physiological pH, have a low molecular weight, and have low binding to serum proteins (Figure 5) [235].

### 4.2. Crossing the Blood-Brain Barrier

Lipophilic, nonionized at physiological pH, and low molecular weight active substances penetrate the Central Nervous System (CNS) [236,237,238]. However, various transport strategies have been developed for the transport of small molecules with poor fat solubility, hydrogen bonding functional groups, and water-soluble active substances such as peptides and proteins to the CNS. These can be classified into three groups: surgical, pharmacologic and physiologic methods. If we briefly explain the content of these methods: opening of tight junctions with osmotic effect, active substance modifications-use of prodrug, use of special transport systems in the brain (CMT), use of polymeric carriers such as nanoparticles and liposomes [210,239]. In the opening of tight junctions with osmotic pressure effect, endothelial cells shrink and tight junctions in the blood brain barrier are temporarily opened by osmotic fluid exchange using solutions of osmotic agents such as mannitol and arabinose in appropriate concentrations. Although prodrugs formed by preparing lipophilic conjugates of active substances with various chemical substances have advantages such as high lipophilic properties that allow very good penetration, transport to the brain, and easy crossing of the lipophilic endothelial barrier, it is an approach that is not very feasible due to the difficulty and high cost of prodrug design [240,241]. Another strategy, CMT, is a potential route for the transport of circulating nutrients and peptides from the blood to the brain. These transport systems are useful systems that reach saturation, show molecular selectivity, and enable the transport of various active substances (small molecules, peptides, etc.) to the brain. Vectors such as chimeric peptides, modified proteins and peptidomimetic monoclonal antibodies are used for drug targeting to the brain. In these systems, a conjugate of the active substance is prepared by binding it to a peptide or monoclonal antibody to take advantage of facilitated transport. While the active substance in the prepared conjugate maintains its biological activity, the MAb binds to the receptor and enables the drug to pass through the BBB via receptor-mediated transport (RMT). Another method is the design of active substance carrier polymeric systems such as nanoparticles and liposomes. The drug is entrapped in the polymer or adsorbed on the surface. In addition, the nanoparticle/active substance formulation is coated with a surfactant (polysorbate 80). The pharmacological effect of the nanoparticles is related to the formulation of these structures. The surfactants used cause the opening of the BBB [242]. In a study, the efficacy of DOX-loaded polysorbate 80-coated nanoparticles against brain tumors was investigated. As a result of chemotherapy studies on rats with glioblastoma, it was determined that polysorbate 80-coated nanoparticles could cross the blood brain barrier and the doxorubicin they carried could reach therapeutic concentrations in the brain (Figure 5C) [243,244].

### 4.3. Cancer Immunotherapy

A diverse array of biomaterials has been employed for the delivery of immunomodulatory substances. In the context of cancer immunotherapy, implantable drug delivery systems can serve two distinct purposes: first, they can be used to administer immunomodulatory agents that target and disrupt checkpoints in the cancer immune cycle, and second, they can facilitate the delivery of cells for adoptive cell transfer (ACT) into cancerous tissue, thereby enhancing the survival and proliferation of these cells. A variety of immunomodulatory drugs, antibodies, antigens, cytokines, and inhibitors have been loaded into implanted biopolymeric drug delivery systems to offer immune-based protection against cancer. Bencherif et al. developed an intelligent DDS in the form of a sponge-like cryogel, where a covalently conjugated RGD peptide was encapsulated alongside cytosine-phosphodiester-guanine oligodeoxynucleotide. This cryogel vaccine was subcutaneously administered to mice and resulted in a potent and sustained antitumor lymphocyte response. In a B16-F10 tumor model, this approach achieved an impressive 80 percent survival rate. Furthermore, the effectiveness of the cryogel vaccines was underscored by the fact that every vaccinated mouse that survived the initial tumor test also withstood a subsequent second test. This highlighted the enduring protective immunity that cryogel vaccinations can provide.

Cancer is one of the most important health problems of our time. In developed countries, new cancer cases are increasing day by day as a result of population growth as well as a Western-style diet, smoking, alcohol consumption and lack of physical activity. Cancer is a cell disease. The human body consists of millions of cells. Our body is constantly producing new cells in order to grow, to replace dead cells, or to repair cells damaged by injury. Cancer, in its shortest definition, is the uncontrolled proliferation of cells. These abnormally proliferating cells invade the tissues and organs where they are located and even far away, causing functional disorders in these areas. The high mortality rate in cancer increases the importance of the subject even more.

For this reason, cancer is one of the most studied topics today, and research continues for more effective and less side-effective treatment. Current methods of cancer treatment include surgery, radiotherapy, chemotherapy and immunotherapy. The most important factor limiting the success of cancer treatment is that anticancer agents used in conventional treatment are not selective for tumor cells and tissues. Almost all chemotherapeutic agents are known to have side effects on normal tissues and organs. Especially anticancer drugs such as cisplatin, cyclophosphamide, methotrexate, mitomycin, and cyclosporine are known to cause severe nephrotoxicity. The main goal of cancer treatment is to destroy the cancer cell without affecting normal tissues. This is possible by selectively targeting the cancer cell. The ideal properties of the drug in tumor targeting can be listed as increasing the localization of the drug in the tumor by active or passive targeting, reducing the accumulation of the drug in non-targeted tissues, minimizing drug leakage from transition zones, protecting the drug from degradation, allowing the drug to remain in the targeted area for the desired period of time, facilitating the uptake of the drug into the cell, and using biocompatible and biodegradable drug carrier system components. For this purpose, interest in different forms of pharmaceutical carrier systems (liposomes, nanoparticles, active substance–polymer conjugates and polymeric micelles, dendrimers) directly binding to the active substance or entrapping the active substance and targeting drugs in this way is increasing day by day. With targeting, conventional, biotechnological and gene-derived drugs can be selectively delivered to specific parts of the body such as organs, tissues and cells. With this selective targeting, undesirable side effects are reduced, the most appropriate therapeutic response is achieved, and substances with toxic effects at high doses can be used safely. New drug carrier systems used for targeting purposes act according to the passive or active targeting principle. Systems such as nanoparticles, microcapsules, microspheres and liposomes, which are prepared using macromolecules such as dextran, albumin, DNA, polyamino acids and other polymers from non-specific carriers, accumulate in the tumor due to environmental characteristics such as tumor size and the EPR effect resulting from the lack of lymphatic drainage around the tumor. In addition, the physicochemical properties of the macromolecule used, such as molecular weight and charge, are also effective in aggregation in the tumor. While these systems can be directed to tumors by passive targeting, they can also be used for active targeting by combining them with molecules such as antibodies, ligands, and peptides.

### 4.4. Molecular Targeted Therapy

Molecular targeted therapy involves anticancer drugs designed to bind to specific molecular targets. These drugs generally work by specifically binding to proteins that play a critical role in tumor development. This approach is more advantageous than conventional cytotoxic chemotherapy. With targeted therapy, host cell toxicity, which is an important problem in cytotoxic chemotherapy, has been largely eliminated. Molecular targeted therapy can be classified into two categories: small molecules (tyrosine kinase inhibitors) and monoclonal antibodies. Small molecules (tyrosine kinase inhibitors) are organic compounds smaller than 800 daltons [246,247,248]. These molecules can easily penetrate the cell membrane. They inhibit tumor cell proliferation, and antiapoptotic effects, angiogenesis and metastasis caused by the tyrosine kinase enzyme. These molecules are named with the suffix “-ib” due to their inhibitory properties. Examples of tyrosine kinase inhibitors are imatinib, gefitinib, and erlotinib. “Imatinib”, used in the treatment of chronic myeloid leukemia (CML), is one of the successful studies of molecular targeting research. The BCR-ABL mutation related to the Philadelphia chromosome has been detected in patients with CML. Studies have shown that imatinib only binds to myeloid cells containing the BCR-ABL mutation and prevents their proliferation, without harming normal cells. Additionally, fewer side effects were observed compared to cytotoxic chemotherapy [249,250]. Monoclonal antibodies are molecules designed from humanized antibodies that bind to cancer cell-specific antigens. Monoclonal antibodies are named with the suffix “-mab”. Monoclonal antibodies such as bevacizumab, cetuximab, panitumumab, rituximab, ranibizumab and transtuzumab used in the treatment of solid tumors have been approved by the FDA [251,252,253,254]. Bevacizumab and ranibizumab target vascular endothelial growth factor (VEGF) (Figure 5D) [245,255,256]. Bevacizumab is used in colorectal cancer [257], non-small cell lung cancer (NSCLC) [258,259], metastatic renal cancer [260,261], and glioblastoma [262,263,264]. Ranibizumab is used in the treatment of diabetic macular edema. Trastuzumab targets the HER2/neu receptor and is used in HER2-positive metastatic breast cancer. Cetuximab targets the epidermal growth factor receptor (EGFR). It is used in the treatment of colorectal cancer and NSCLC. Rituximab targets CD20 on B cells and is used in non-Hodgkin lymphoma. Another application of monoclonal antibodies is their use as a drug carrier system in the form of an antibody–drug conjugate (ADC). The monoclonal antibody binds to the cancer cell. The antibody–cytotoxic drug conjugate passes through the intracellular membrane of the tumor cell, resulting in cell death. This technology provides a wide therapeutic range by targeting only cancer cells, thereby minimizing the potential side effects of the cytotoxic drug. In 2013, ado-trastuzumab emtansine (T-DM1) was approved by the FDA for HER2-positive metastatic breast cancer. In similar work, an antibody–drug conjugate (ADC) has been developed for the treatment of breast cancer (Figure 5E) [265].

**Table 2:** Complex neurological disorders and drug delivery methods.

Complex Neurological Disorder	Drug Delivery Methods	Description	Ref.
Epilepsy	Electrophoretic drug delivery	The microfluidic ion pump facilitates the tailored delivery of inhibitory neurotransmitters by detecting seizure activity and transporting ions via the ion exchange membrane via electrophoresis. Mice have been used to test this strategy.	[266,267]
	Implanted intracerebroventricular delivery system	For patients with epilepsy, the device delivers valproic acid, an anti-seizure medicine, into their cerebrospinal fluid for a protracted course of treatment.	[268]
	Microencapsulation of anti-seizure medications	Polymer cores containing lacosamide, an anti-seizure medication, are enveloped by drug-free polymer shells and have been examined in vitro using synthetic cerebrospinal fluid.	[269]
	Nanoparticles	Gold nanoparticles coated with glucose are linked to the anti-seizure medication lacosamide, intended for intravenous delivery in rats.	[270]
Stroke	Liposome	ZL006, a neuroprotectant and nNOS/PSD-95 inhibitor, was injected into T7-conjugated PEGylated liposomes in stroke models in living rats and mice.	[271,272]
Brain Cancer	Bioresorbable electronic patch	In a mouse model of brain tumor, the patch promotes prolonged drug release and improves drug penetration by modest heat activation	[273]
	Nanoparticles	Dasatinib, an anti-cancer medication, was administered to a mouse model of glioblastoma using Cornell prime dots conjugated with αv integrin-binding/nontargeting peptides and tagged with PET (positron emission tomography) labels.	[274]
Traumatic Brain Injury	Exosomes	Intravenous delivery of mesenchymal stem cell (MSC)-derived exosomes, which contain physiologically active molecules and reduce inflammation in traumatic brain injury (TBI), has been shown to be effective. Animal studies have shown that these exosomes are capable of crossing the blood-brain barrier.	[275,276]
	Nanoparticles	Poly(lactic-co-glycolic acid) nanoparticles were used to treat traumatic brain injury (TBI) in vivo in mice by delivering siRNA. These polysorbate 80-coated nanoparticles promoted receptor-mediated transport across the lipoprotein receptor.	[277]
Alzheimer’s Disease	Magnetic resonance-guided low-intensity focused ultrasound	Significantly more of the blood-brain barrier can be reversibly opened when magnetic resonance-guided low-intensity focused ultrasound is applied to the human entorhinal cortex and hippocampal regions.	[278]
Parkinson’s Disease	Supramolecular gel	A hydrogel containing the amino acid L-DOPA demonstrates swift drug release upon intranasal delivery in mice.	[279]
	Nanoparticles	Protocells, carrying both Parkinson’s disease drugs, levodopa and curcumin, had their lipid bilayer modified for brain targeting. This modification was achieved through intraperitoneal injection in a mouse model of Parkinson’s disease.	[280]
	Oral and maxillofacial device	A system implanted in the oral or maxillofacial region is specifically engineered to transport drugs to the brain via the respiratory mucosa. This functionality was evaluated through testing in a live rabbit model.	[281]

## 5. Advantages of Drug Targeting

Targeted drug delivery systems have revolutionized the pharmacological landscape by enabling the transport of active substances directly to pathological regions or specific cells, thereby reducing the required dosage and minimizing side effects [282,283,284]. These systems allow active compounds to access previously unreachable sites, such as intracellular regions or pathogens like viruses, bacteria, and parasites. By utilizing pharmacological receptors, targeted delivery ensures that the drug remains inert until it reaches the site of action, optimizing dosing frequency and therapeutic speed. Consequently, drug administration protocols are simplified, therapeutic efficiency is enhanced, and treatment costs are reduced. Selective drug transport offers two critical benefits: ensuring optimal drug efficacy by delivering the active compound to the intended site at the desired rate, and reducing systemic distribution to minimize side effects. By restricting the distribution of the drug to target organs, site-specific delivery significantly improves the therapeutic index. This approach holds substantial promise for managing conditions such as uncontrollable intracellular infections (e.g., HIV/AIDS), central nervous system disorders, immune system diseases, cancer, and cardiovascular pathologies [284,285,286,287,288].

The design and development of targeted drug delivery systems presents both opportunities and challenges, necessitating a thorough understanding of four fundamental components: the drug, the target, the disease, and the carrier system. The efficacy of these systems depends on identifying mechanisms to enhance selectivity, which may be biochemical, physiological, or immunological in nature. This requires a multidisciplinary approach, integrating expertise across various scientific domains. Traditional drug administration methods, such as oral or intravenous delivery, often result in widespread distribution of the drug, leading to off-target effects and unwanted side reactions. Targeted drug delivery, by contrast, focuses on directing drug molecules to specific receptors in the desired tissues or regions [3,289,290]. This precision not only enhances the pharmacological response but also offers significant clinical benefits by minimizing adverse effects and maximizing therapeutic outcomes.

## 6. Drug Market Used in Cancer Nanotechnology

Despite considerable advances in medicine and technology, cancer continues to claim the lives of millions each year [291,292]. Extensive study over decades highlights the disease’s ever-changing character. While treatment choices have improved, the long-term problem is the significant adverse effects of strong chemotherapies [293,294]. The widespread establishment of resistance mechanisms is a key impediment to successful cancer therapies. When the primary cancer-causing pathways are shut down, other signaling pathways are activated, allowing the cancer to adapt and survive [295,296]. This adaptive capability poses a significant challenge in effectively treating cancer.

The approach to cancer therapy must shift from focusing only on novel medicines to improving existing treatments and diagnostics using creative, effective, and practical ways. Pain affects 55% of cancer patients under therapy and 66% of those in late stages [297]. Chemotherapies without specific targeting mechanisms attack both malignant and healthy cells indiscriminately, resulting in systemic toxicity that impairs patients’ quality of life [298,299]. Furthermore, the benefits of early detection are obvious. Early stage cancers have much greater 5-year survival rates, lower total patient expenses, and often require less aggressive treatment options [300,301]. This highlights the crucial role of early identification in improving outcomes and reducing the impact of cancer on patients’ lives. Nanotechnology might provide a viable answer by improving the targeting capabilities of current medicines. It has the potential to improve the effectiveness of localized medication delivery and thereby reduce systemic toxicity. Furthermore, nanotechnology has the potential to increase diagnostic sensitivity, imaging methods, and radiation treatment [302,303]. It has the possibility of changing several areas of cancer treatment and detection by utilizing the unique capabilities of nanotechnology, such as precision targeting and controlled release.

There are many cancer types, and every cancer type possesses different costs for its treatments. Simioa Chen et al. [304] evaluated the global economic cost of different cancer types from 2020 to 2050. According to their study, cancer is expected to cost the world economy $25.2 trillion in international currencies from 2020 to 2050. This amount is comparable to a 0.55% yearly tax on world GDP. Tracheal, bronchus, and lung cancer (15.4%); colon and rectum cancer (10.9%); breast cancer (7.7%); liver cancer (6.5%); and leukemia (6.3%) are the malignancies with the biggest economic expenses. Furthermore, in high-income nations, treatment expenditures contribute more to the overall economic burden of cancer than in low-income countries. This suggests that the costs of cancer treatment have a greater influence on the total economic cost in high-income countries. China and the United States have the greatest economic costs from cancer, accounting for 24.1% and 20.8% of the total worldwide burden, respectively. Despite the fact that low- and middle-income nations account for 75.1% of cancer-related mortality, they account for 49.5% of cancer-related economic losses.

The integration of nanotechnology into medicine and pharmacy has profoundly transformed drug development and delivery strategies. Nano-formulated drugs and nano-drug delivery systems represent innovative technologies characterized by their ability to achieve highly precise targeting, enhance drug bioavailability, and minimize adverse effects. These advancements are particularly evident in the treatment of cancer, neurodegenerative disorders, cardiovascular diseases, and infectious diseases, underscoring the significant medical potential of such systems. This article reviews the current state of nano-formulated drugs, highlighting examples from clinical trials and approved products, alongside their applications in various diseases. Nano-formulated drugs are pharmaceutical products engineered at the nanoscale (1–100 nm). These systems offer several distinct advantages, including targeted delivery, improved bioavailability, enhanced permeability and retention (EPR) effects, controlled release, and multifunctional integration. Nano-drug delivery systems leverage diverse carrier platforms, such as liposomes, polymeric nanoparticles, micelles, metallic nanoparticles, and dendrimers. Notable examples of clinically successful nano-drugs include Doxil (doxorubicin-loaded liposomes), Abraxane (paclitaxel-loaded polymeric nanoparticles), and Genexol-PM (paclitaxel-loaded micelles), which have significantly advanced therapeutic efficacy and patient outcomes. Nano-formulated drugs have contributed numerous innovative breakthroughs to medical science and patient care. In oncology, these systems have demonstrated the capacity to precisely target the tumor microenvironment, reducing systemic side effects while improving therapeutic efficacy compared to conventional chemotherapeutics. Furthermore, theranostic applications allow nanoparticles to serve dual roles in diagnosis and therapy. For instance, gold nanoparticles are widely utilized in the imaging and treatment of cancerous tumors. In the field of infectious diseases, nanoformulations provide a highly targeted and effective approach to combat antibiotic-resistant pathogens. Additionally, the development of nano-drugs capable of traversing the blood–brain barrier represents a significant advancement, offering promising therapeutic options for neurodegenerative diseases such as Alzheimer’s and Parkinson’s disease. In conclusion, nano-drug delivery systems occupy a critical role in both current clinical practice and research endeavors. Their modular and customizable nature allows for adaptation to a wide array of therapeutic needs, providing innovative solutions for complex medical challenges. Current clinical trials and the availability of approved nano-drug products underscore the transformative potential of nanotechnology in modern medicine, paving the way for future advancements in patient care. Nano-formulated drugs, their stages, and applications in cancer treatment were given in Table 3.

**Table 3:** Nano-formulated drugs, their stages, and applications in cancer treatment.

Product	Type	Indication	Stage	Mechanism/Advantages
Doxil [305,306,307]	Liposomal formulation	Ovarian and breast cancer	Marketed	Encapsulates doxorubicin, reduces cardiotoxicity, and enhances tumor targeting through EPR effect.
Abraxane [308,309,310]	Polymeric nanoparticles	Pancreatic and breast cancer	Marketed	Albumin-bound paclitaxel improves solubility, bioavailability, and tumor-specific accumulation.
Genexol-PM [309,311,312]	Micellar formulation	Breast cancer	Marketed	Paclitaxel-loaded micelles enhance solubility and reduce side effects compared to conventional forms.
Onivyde [313,314,315]	Liposomal formulation	Pancreatic cancer	Marketed	Irinotecan encapsulation increases circulation time and targets tumors via passive accumulation.
BIND-014 [316,317]	Targeted nanoparticles	Prostate cancer	Clinical trials	Docetaxel-loaded polymeric nanoparticles with ligand-based targeting to PSMA receptors on cancer cells.
Nanoxel [309,318,319]	Polymeric nanoparticles	Breast and ovarian cancer	Marketed	Nanoparticle-based paclitaxel improves drug delivery efficiency and reduces hypersensitivity reactions.

Nano-formulated drugs represent a significant advancement in cancer therapy, offering solutions to overcome the limitations of conventional treatment approaches [320,321,322]. By utilizing the potential of nanotechnology, these drugs are designed to enhance drug bioavailability, improve targeting specificity, and reduce systemic toxicity, thereby optimizing therapeutic outcomes (Table 3). Nano-formulations utilize drug delivery systems that are engineered at the nanoscale to increase the solubility of poorly water-soluble drugs, ensure sustained release, and facilitate targeted drug delivery to cancerous tissues, all while minimizing the impact on healthy cells. This approach is particularly promising in cancer treatment, where precise targeting of tumor cells and the reduction of side effects are critical for improving patient outcomes (Table 4). One of the key features of nano-formulated drugs is their ability to provide controlled drug release, which allows for a more consistent therapeutic effect and reduces the frequency of drug administration. Additionally, nano-carriers, such as liposomes, micelles, and polymeric nanoparticles, play a crucial role in overcoming the challenges associated with drug solubility and stability, thereby enhancing the overall effectiveness of chemotherapy. These formulations also provide the added advantage of reducing the drug’s toxic effects on healthy tissues, which is a common concern with traditional chemotherapy. Among the various nano-formulated drugs, Doxil, a liposomal formulation containing doxorubicin, has gained considerable attention for its ability to deliver the drug to the tumor site more effectively while minimizing cardiotoxicity. Liposomal drug delivery systems enhance the circulation time of the drug and enable more specific targeting of cancer cells, making Doxil a valuable option in the treatment of ovarian and breast cancers [323,324,325]. Similarly, Abraxane, which utilizes polymeric nanoparticles to encapsulate paclitaxel, enhances the drug’s bioavailability and therapeutic efficacy. By overcoming solubility issues and providing a controlled release mechanism, Abraxane has shown promise in the treatment of pancreatic and breast cancers, where conventional paclitaxel formulations often fall short [326,327,328].

Another important nano-formulation, Genexol-PM, employs micellar technology to improve the solubility of paclitaxel and reduce side effects [329,330,331,332]. This formulation has demonstrated improved therapeutic outcomes in the treatment of breast cancer, offering an alternative to traditional paclitaxel formulations that are associated with high toxicity and limited solubility. Onivyde, a liposomal formulation of irinotecan, is specifically designed for the treatment of pancreatic cancer. By encapsulating irinotecan in liposomes, this formulation prolongs the drug’s half-life and allows for more efficient delivery to the tumor, thereby improving treatment efficacy and reducing systemic toxicity [333,334,335,336].

In addition to these formulations, BIND-014, a targeted nanoparticle delivery system encapsulating docetaxel, represents a breakthrough in the treatment of prostate cancer. This drug delivery system is designed to selectively target cancer cells, reducing the exposure of healthy cells to the toxic effects of chemotherapy. The use of targeting ligands allows for the preferential accumulation of the drug in tumor tissues, enhancing therapeutic outcomes while minimizing off-target effects [316,337,338]. Similarly, Nanoxel, another polymeric nanoparticle-based formulation of paclitaxel, has shown efficacy in the treatment of both breast and ovarian cancers. By improving the solubility and bioavailability of paclitaxel, Nanoxel ensures that higher concentrations of the drug reach the cancer cells, thereby increasing the treatment’s effectiveness and reducing side effects [318,319].

These nano-formulations exemplify the significant strides made in the development of targeted cancer therapies. Each drug formulation utilizes different nanocarriers and drug release mechanisms to optimize the delivery of chemotherapy agents to tumor cells while minimizing adverse effects on healthy tissues. The ability to engineer drugs at the nanoscale offers numerous advantages, including improved drug solubility, sustained release, and more precise targeting, all of which contribute to enhanced treatment outcomes and reduced side effects. The clinical stages of these nano-formulated drugs demonstrate their potential to revolutionize cancer therapy. While some of these drugs, such as Doxil, Abraxane, and Nanoxel, have already been successfully marketed and are widely used in clinical practice, others, such as BIND-014, are still undergoing clinical trials. As these therapies continue to evolve, they hold the promise of offering more effective, targeted, and personalized treatment options for cancer patients.

In conclusion, nano-formulated drugs represent a paradigm shift in cancer treatment, offering targeted and controlled drug delivery that enhances therapeutic efficacy while reducing harmful side effects. The development of these innovative therapies has the potential to significantly improve the treatment landscape for various cancer types, providing new hope for patients who may not have responded to traditional treatment options. Continued research and clinical trials are essential to fully realize the potential of nano-formulations in oncology, with the goal of making these advanced therapies accessible to a broader patient population and further advancing the field of cancer treatment.

**Table 4:** Drugs used in hydrogel drug delivery systems and their effects.

Commercial Name of Drug	Loaded Drug	Carrier Polymer of Hydrogel	Results	Limitations	Ref.
-	Insulin	Chitosan	Generally, 0.5% of orally administered insulin reaches the bloodstream.	Enzymatic barriers and degradation due to the highly acidic environment in the stomach. Onlay a small amount of insulin that is administered reaches the bloodstream due to its hydrophilicity and large size. Also, the structure of intestine is not suitable because of the monolayer of intestinal cells.	[339]
Sigma Chem. Co., (St. Louis, MO, USA)	Chlorhexidine gluconate	Chitosan films (partially deacetylated chitin)	A 2% chitosan gel was shown to have a higher viscosity than a 1% gel, making it more appropriate for topical administration without compromising spreadability. Furthermore, over the course of three hours, the 2% gel formulation showed a greater release of chlorhexidine (Chx), according to in vitro release experiments.	As an oral rinse, chlorhexidine (Chx) has been shown to be effective. Gels, on the other hand, have the ability to significantly lengthen their residence duration in the oral cavity, which might boost their therapeutic efficacy in comparison to solutions. In this study, chitosan was used as a carrier to create gel and film formulations that delivered Chx into the oral cavity. Because of its high viscosity and bioadhesive properties, chitosan gel is expected to remain in the oral cavity for an extended period of time, allowing for sustained drug release and improving clinical efficacy.	[340]
-	Vascular endothelial growth factor-165 (VEGF) proangiogenic gene	Graphene Oxide-Based Hydrogel	This method has several significant benefits over the widely studied stem cell treatment. The main obstacles to stem cell therapy are frequently occuring immune rejections, difficulties preserving cell viability and retention at the target site, possible teratoma formation risks, and a plethora of ethical, practical, and technical difficulties with cell isolation and culturing. This cooperation of graphene oxide/DNA therapy and tissue engineering can be a novel strategy for regenerative medicine.	The application of intravenous drug delivery of graphene oxide is able to increase the rate of reactive oxygen and induced mutagenesis. In this case, the long-term effects of graphene oxide should be further investigated.	[341]
-	Chlorpromazine (antipsychotic drug)	Chitosan/Pectin	This study’s results imply that chitosan/pectin polyelectrolyte complexes can be used to create mucoadhesive nasal inserts with different medication release properties. It was able to modify the inserts’ water absorption behavior as well as the release and penetration of chlorpromazine hydrochloride at the administration site by carefully selecting the chitosan/pectin molar ratio during complex manufacture.	-	[342]
GlaxoSmithKline, (London, UK)	Betamethasone-17-valerat	Sodium-Deoxycholate	During the study, sodium deoxycholate gels showed substantially more edema inhibition than a commercial cream. Histology experiments further showed that sodium deoxycholate gel did not cause skin irritation. As a result, topical administration of betamethasone valerate (BMV) in the Na-DOC gel formulation appears to be a viable substitute approach.	There is no specification limit for the hydrogel	[343]
-	Dexamethasone	Tyramine-Modified Hyaluronic Acid	In both in vitro and in vivo investigations, the hydrogels made of hyaluronic acid (HA) and tyrosine (Tyr), which encapsulated DMT as a typical anti-inflammatory medication, showed a continuous and extended release of DMT for as long as one month. The study found that the combination of hyaluronic acid (HA) and tyrosine (Tyr) with horseradish peroxidase (HRP) showed some efficacy in the treatment of rheumatoid arthritis (RA). On the other hand, injured cartilage recovered almost entirely when HA–Tyr hydrogels containing DMT were used.	-	[344]
Nanjing Zelang Medical Technology Co., Ltd. (Nanjing, China)	Curcumin	Pluronic F127 and Poloxamer 188	A thermosensitive nasal in situ gel containing curcumin was developed in this study. It had desirable qualities such as a short gelation time, prolonged drug release, and safe biological properties. When compared to intravenous (i.v.) treatment, the nasal in situ gel boosted curcumin absorption in the brain. Notably, the fluid-like fluidity of this in situ gel prior to contact with the nasal mucosa is the primary benefit of this in situ gel over a traditional gel. This feature facilitates patient administration and assures correct medicine dose.	-	[345]
Riyadh Pharma (Riyadh, Saudi Arabia)	Acyclovir	Polyvinylpyrrolidone (PVP)	The release properties of hydrogels showed that PVP gels had higher release rates, and that this rate increased even more when PEG or glycerol was included. Histopathological analyses confirmed PVP hydrogel’s safety for mucosal administration.	In gel formulations containing PEG, nasal mucosal injury was less severe than in formulations including glycerol.	[346]
Janssen-Cilag SpA Co. (Latina, Italy)	Tramadol	Poloxamer	In terms of gelling behavior, drug content, and drug release, the formulation for thermosensitive gelling showed reliable batch-to-batch consistency. When a membrane was added to the immersion cell, the medication released more slowly than when it was not. Moreover, a quicker drug release was connected with a greater paddle rotation speed. Notably, the related drug release efficiency (DE) values and membrane thickness showed a strong and substantial linear connection.	Moreover, an in-depth understanding of the testing parameters that have to be set during dissolving tests is required due to the growing interest in thermosensitive hydrogels for parenteral usage.	[347]
-	Calcium phosphate-DNA	Alginate	In this work, transplanted preosteoblasts were given calcium phosphate-DNA (CaP-DNA) via alginate hydrogels to enhance the process of bone formation. Alginate hydrogels are also a flexible biomaterial delivery platform that allows for the modification of the scaffold’s physical and biological characteristics. This involves modifying the mechanical characteristics, degradation profile, and adhesiveness of the cell.	-	[348]
	GFP-expressing plasmid	Poly(L-lactide)-b-poly(ethylene glycol) (FA-PEG-PLLA)	Activated macrophages internalize FA-PEG-PLLA by means of folate receptor-mediated endocytosis. When their hydrogels release intact 3LM, primary macrophage transfection is facilitated effectively. Because of this, folic acid—3-layer micelles have the potential to be a revolutionary therapeutic for rheumatoid arthritis when developed as an in situ gel. They also show promise as a delivery method for receptor-mediated drug or gene delivery.	-	[349]

## 7. Summary

The recent advancements in biopolymer drug delivery applications and nanomedicine have been reviewed. Due to their extensive control over the release of bioactive substances, biopolymeric drug delivery systems have demonstrated tremendous promise in the treatment of numerous diseases. These DDSs can provide passive or active drug delivery and can be constructed from a variety of biopolymers. Additionally, they do not have to be implanted using invasive surgery at the diseased area, as is the case with injectable DDSs, which may simply be injected in the target place to prevent subsequent issues from procedures. But polymeric systems are yet unable to treat complicated diseases. So, they must be utilized in concert with nanoparticulate systems to treat diseases like cancer. The integration of cancer therapy and diagnosis has received a lot of attention in recent years as nanomedicine has advanced. Nanomedicine has already transformed how we locate and use medications in biological systems. Nanomedicine advancements have enabled us to identify diseases and, in some cases, pair therapy with a diagnosis.

Rapid developments continue to occur in the pharmaceutical industry in the 21st century. Thanks to advances in biotechnology and nanotechnology, new drugs with protein and nucleic acid structures are being produced. These molecules are expected to be more effective in binding to the target receptor, thanks to studies on improving their clinical properties with the help of nanocarriers. Studies are ongoing to selectively deliver conventional, biotechnological and gene-derived drugs to specific parts of the body such as organs, tissues and cells through drug targeting. It is aimed to prevent undesirable effects by applying cytotoxic drugs, especially those used in cancer diagnosis and treatment, in the form of nanocarriers. The use of molecular targeted therapy in cancer treatment provides successful results. Studies initiated under the name “Cancer Genome Atlas Project” aiming to find personalized drug targets are continuing. With this project, people’s gene maps can be created, individual differences in cancer risk and tumor structure can be detected, so that specific drugs can be developed. In the treatment of central nervous system diseases, nanocarriers are being developed to cross the blood-brain barrier, increase the bioavailability of the drug and be effective in the desired region. In the treatment of cardiovascular diseases, targeting studies are carried out for the factors that cause the disease. In this way, it is expected to prevent myocardial infarction, which is the leading cause of death in the world. In order to prevent the undesirable effects of drugs used in the treatment of rheumatoid arthritis, especially glucocorticoids and methotrexate, studies are being carried out to administer these drugs with the help of nanocarriers, aiming for the drug to act only in the inflamed area (pannus). Additionally, drugs that can specifically bind to the receptors of molecules that cause rheumatoid arthritis are being developed. The acceleration of developments in drug targeting studies is promising for the treatment of important uncontrolled diseases. As the molecular and cellular biology of diseases is better understood; Thanks to the discovery of new targets and new ligands, it is expected that the drug will be transported to the targeted area more effectively and cause fewer side effects. Thus, the treatment of diseases can be done more rationally and effectively.

## Data Availability

No new data were created or analyzed in this study. Data sharing is not applicable to this article.
